# The dynamic role of soluble urokinase plasminogen activator receptor (suPAR) in monitoring coagulation dysfunction during COVID-19 progression: a review

**DOI:** 10.1097/MS9.0000000000002791

**Published:** 2024-12-19

**Authors:** Emmanuel Ifeanyi Obeagu

**Affiliations:** Department of Biomedical and Laboratory Science, Africa University, Zimbabwe

**Keywords:** biomarker, coagulation disorders, COVID-19, suPAR, thrombosis

## Abstract

COVID-19, caused by the severe acute respiratory syndrome coronavirus 2, has led to significant morbidity and mortality worldwide, with severe complications often involving coagulation dysfunction and thromboembolic events. Identifying reliable biomarkers for early detection and monitoring of these complications is crucial for improving patient outcomes. The soluble urokinase plasminogen activator receptor (suPAR) has emerged as a potential biomarker in this context, given its role in inflammation and immune response. Elevated suPAR levels correlate with disease severity and inflammatory markers, making it a valuable indicator of the complex interplay between inflammation and coagulation observed in COVID-19. Elevated suPAR levels have been linked to an increased risk of thromboembolic events, such as deep vein thrombosis and pulmonary embolism. Combining suPAR measurement with other coagulation markers, such as D-dimer, enhances the predictive accuracy for thrombotic complications. Furthermore, higher suPAR levels are associated with increased disease severity, intensive care requirements, and higher mortality rates, underscoring its significance in risk stratification and therapeutic decision-making. The integration of suPAR measurement into routine clinical practice for COVID-19 could significantly aid in early diagnosis, risk assessment, and monitoring of therapeutic interventions. By providing insights into the patient’s inflammatory and coagulation status, suPAR can guide the timely initiation of anticoagulant therapy and other treatments aimed at reducing thromboembolic complications. As research continues to validate suPAR’s utility across diverse populations and clinical settings, it holds promise for becoming an integral component of clinical management strategies to mitigate the morbidity and mortality associated with COVID-19.

## Introduction

COVID-19, caused by the severe acute respiratory syndrome coronavirus 2 (SARS-CoV-2), has rapidly evolved into a pandemic, affecting millions of individuals worldwide. Since its emergence in late 2019, COVID-19 has imposed unprecedented strains on health care systems due to its high transmission rate and significant morbidity and mortality. The clinical presentation of COVID-19 ranges from mild respiratory symptoms to severe pneumonia, acute respiratory distress syndrome, multi-organ failure, and death^[1,2]^. One of the most concerning complications associated with severe COVID-19 is coagulation dysfunction, which frequently leads to thromboembolic events. These events include deep vein thrombosis, pulmonary embolism, and microthrombi in various organs, contributing significantly to the high mortality rates observed in critically ill patients^[3,4]^. The pathophysiology of coagulation dysfunction in COVID-19 is complex and involves a hyperinflammatory response, often referred to as a “cytokine storm.” This exaggerated immune response triggers endothelial damage, activation of the coagulation cascade, and suppression of fibrinolysis, leading to a prothrombotic state. Elevated levels of inflammatory cytokines such as interleukin-6 (IL-6) and tumor necrosis factor-alpha are common in severe COVID-19 and are associated with increased thrombotic risk^[5,6]^. Given the critical role of coagulation dysfunction in COVID-19 morbidity and mortality, there is an urgent need for reliable biomarkers that can provide early detection and monitoring of these complications. Such biomarkers would enable timely intervention, improve risk stratification, and guide therapeutic decisions, ultimately enhancing patient outcomes and reducing health care burdens^[7,8]^.

The soluble urokinase plasminogen activator receptor (suPAR) has recently gained attention as a potential biomarker for inflammation and coagulation disorders. suPAR is a circulating form of the urokinase plasminogen activator receptor (uPAR), which is involved in various physiological processes including cell migration, tissue remodeling, and immune responses. Elevated suPAR levels have been associated with adverse outcomes in several diseases, including infections, cardiovascular diseases, and more recently, COVID-19^[9,10].^ suPAR is released by activated immune cells, including neutrophils, monocytes, and macrophages, in response to inflammatory stimuli. In COVID-19, suPAR levels have been found to correlate with the severity of the inflammatory response and disease progression. This makes suPAR a valuable marker for assessing the extent of inflammation and potential complications arising from the immune response in COVID-19 patients^[11,12].^ Beyond its role in inflammation, suPAR has been implicated in coagulation disorders. suPAR can influence fibrinolysis, the process by which blood clots are broken down, thus playing a role in maintaining the balance between coagulation and bleeding. In COVID-19, elevated suPAR levels have been linked to increased coagulation activity and a higher risk of thromboembolic events, underscoring its potential as a marker for coagulation dysfunction^[13,14]^. The diagnostic value of suPAR in COVID-19 lies in its ability to provide early indications of both inflammation and coagulation abnormalities. Measuring suPAR levels can help identify patients at higher risk of developing severe complications, including thromboembolic events, thereby enabling early and targeted therapeutic interventions. Studies have shown that suPAR levels, when combined with other biomarkers like D-dimer, enhance the predictive accuracy for thrombotic complications[15]. In addition to its diagnostic capabilities, suPAR has significant prognostic value. Higher suPAR levels have been associated with increased disease severity, greater likelihood of requiring intensive care, and higher mortality rates. Monitoring suPAR levels throughout the course of the disease can provide valuable insights into the patient’s prognosis, guiding clinical management and resource allocation[14].

This review aims to provide a comprehensive analysis of the role of suPAR in monitoring coagulation dysfunction during the progression of COVID-19.

## suPAR: overview and mechanisms

suPAR, short for soluble urokinase plasminogen activator receptor, is a circulating protein that holds a multifaceted role in various physiological and pathological processes. Its relevance in the context of COVID-19-associated coagulopathies stems from its involvement in inflammation, immune responses, endothelial function, and coagulation regulation[16]. The uPAR is a glycosylphosphatidylinositol-anchored membrane protein expressed on various cell types, including immune cells, endothelial cells, and certain cancer cells. suPAR is a cleaved form of uPAR that is released into circulation. While its precise physiological functions are still under investigation, suPAR is thought to modulate immune responses, cell adhesion, migration, and tissue repair processes[17].

suPAR is produced by proteolytic cleavage of uPAR or as an alternatively spliced variant. It is released into the bloodstream and can be detected in serum or plasma. Its levels can be influenced by various factors, including inflammation, infection, and tissue injury. Elevated suPAR levels have been observed in several pathological conditions and are considered indicative of systemic immune activation and cellular damage[16]. suPAR acts as a chemoattractant for immune cells, promoting their recruitment to sites of inflammation. It interacts with various immune receptors, including formyl peptide receptors, integrins, and chemokine receptors, influencing immune cell migration, activation, and cytokine production. suPAR is implicated in perpetuating inflammatory responses, contributing to the pathogenesis of inflammatory diseases[17]. suPAR has been associated with endothelial dysfunction, disrupting the delicate balance of the endothelium[18]. It affects endothelial cell adhesion, permeability, and angiogenesis. Elevated suPAR levels are linked to impaired endothelial function, potentially contributing to vascular pathology, thrombosis, and organ damage. Emerging evidence suggests a relationship between suPAR and coagulation pathways. suPAR may influence the coagulation cascade by interacting with components of the fibrinolytic system and modulating endothelial activation^[19,20]^. Its involvement in promoting a prothrombotic state in certain pathological conditions, including COVID-19, is being investigated.

## COVID-19-associated coagulopathies

COVID-19, caused by the novel coronavirus SARS-CoV-2, has been associated with a spectrum of coagulation abnormalities that significantly contribute to disease severity and complications. These coagulopathies range from mild hemostatic alterations to severe disseminated intravascular coagulation (DIC) and thrombotic events, posing challenges in clinical management and patient outcomes^[21–25]^. COVID-19 patients frequently exhibit a state of hypercoagulability characterized by elevated levels of fibrinogen, D-dimers, and other markers of coagulation activation. This hypercoagulable state predisposes individuals to an increased risk of thrombotic events, including venous thromboembolism, pulmonary embolism, deep vein thrombosis, and arterial thrombosis. Such complications have been reported even in patients receiving anticoagulant therapy^[26–28]^. Severe cases of COVID-19 can progress to DIC, a life-threatening condition characterized by widespread activation of coagulation leading to microvascular thrombosis, consumption of clotting factors, and subsequent bleeding diathesis[29]. The dysregulated coagulation and fibrinolysis cascade observed in DIC contribute to multi-organ dysfunction and increased mortality.

The involvement of microvascular thrombosis, particularly in the lungs and other organs, has been reported in COVID-19 patients. These microthrombi contribute to respiratory compromise, tissue ischemia, and organ dysfunction, further complicating the clinical course of the disease. SARS-CoV-2 can directly infect endothelial cells, leading to endothelial dysfunction and vascular damage. This endothelial involvement is implicated in promoting a prothrombotic state, inflammatory responses, and compromised microcirculatory function. Coagulation abnormalities in COVID-19 have been associated with disease severity, progression to critical illness, and adverse outcomes, including higher mortality rates[30]. Elevated D-dimer levels, a marker of fibrinolysis and clot breakdown, have been consistently linked to increased disease severity and mortality[31].

## Role of suPAR in COVID-19 coagulation disorders

The involvement of suPAR in COVID-19-associated coagulation disorders has garnered significant attention due to its potential role as a biomarker for disease severity and its implications in the underlying pathophysiology. Numerous studies have reported elevated suPAR levels in severe COVID-19 cases. Higher suPAR concentrations have been associated with increased disease severity, progression to critical illness, and adverse outcomes, including mortality. This suggests a potential link between suPAR levels and the severity of coagulation disorders observed in COVID-19 patients^[19,32]^. suPAR has shown promise as a predictive biomarker for the development of coagulation abnormalities in COVID-19. Elevated suPAR levels early in the disease course have been correlated with subsequent thrombotic events, DIC, and other coagulopathies, indicating its potential utility in identifying individuals at higher risk[33]. suPAR is implicated in endothelial dysfunction, which plays a pivotal role in the development of coagulation disturbances in COVID-19. Elevated suPAR levels are associated with impaired endothelial function, disruption of the endothelial barrier, and modulation of vascular tone, contributing to a prothrombotic milieu^[31]^. suPAR’s involvement in inflammation and immune activation may also contribute to coagulation dysregulation. Its ability to influence immune cell migration, cytokine production, and endothelial activation suggests a potential indirect role in the amplification of the inflammatory response leading to coagulation abnormalities in COVID-19[19].

## suPAR as a prognostic marker in COVID-19

suPAR has emerged as a promising prognostic marker in the context of COVID-19 due to its association with disease severity, progression, and adverse outcomes[30]. Elevated suPAR levels have been consistently linked to increased disease severity in COVID-19 patients. Numerous studies have demonstrated a positive correlation between higher suPAR concentrations and more severe clinical presentations, including respiratory compromise, multi-organ dysfunction, and the need for intensive care unit (ICU) admission^[32]^. suPAR levels serve as predictive indicators of adverse outcomes, including mortality, in individuals with COVID-19. Patients presenting with elevated suPAR levels upon admission or during the early stages of the disease are more likely to experience unfavorable clinical outcomes, higher mortality rates, and increased risk of complications such as thrombotic events[33].

suPAR measurement offers a potential tool for risk stratification, aiding clinicians in identifying individuals at higher risk of experiencing severe disease progression and adverse outcomes. It assists in triaging patients, optimizing resource allocation, and guiding appropriate clinical interventions, including close monitoring and aggressive therapeutic approaches for high-risk individuals[32]. suPAR’s prognostic significance extends beyond conventional biomarkers like D-dimers and inflammatory markers. Its independent predictive ability for severe disease progression and adverse outcomes provides complementary information to existing clinical and laboratory parameters, enhancing the prognostic accuracy for COVID-19 patients^[31]^. Identification of high suPAR levels early in the disease course may prompt clinicians to consider more aggressive therapeutic interventions and targeted management strategies tailored to individuals at higher risk of severe complications, thereby potentially improving patient outcomes.

## Therapeutic implications and future perspectives

The therapeutic implications of suPAR in the context of COVID-19 is multifaceted and hold potential implications for guiding therapeutic strategies, prognosis, and future research avenues. Modulating suPAR-associated pathways or downstream effectors might hold potential in mitigating coagulation abnormalities and inflammatory responses, potentially improving clinical outcomes in affected individuals[19]. Further research aimed at developing therapeutic interventions specifically targeting suPAR pathways or its interactions could provide novel treatment modalities for managing COVID-19-associated coagulopathies. These may include pharmacological agents or biologics that modulate suPAR levels or its downstream signaling pathways to attenuate inflammation and coagulation disturbances.

Incorporating suPAR measurements into clinical trials and treatment protocols for COVID-19 could offer insights into its predictive value and response to targeted interventions. Investigating the impact of therapies aimed at modulating suPAR levels or its associated pathways on disease progression, coagulation status, and clinical outcomes may be instrumental in refining treatment strategies. suPAR, as a biomarker, may contribute to personalized medicine approaches in COVID-19. Tailoring treatments based on suPAR levels and associated risk profiles could enable more precise and individualized therapeutic strategies, optimizing patient care and improving prognostication[31]. Longitudinal studies assessing suPAR kinetics over the disease course could elucidate its dynamic changes and prognostic implications at different stages of COVID-19. Furthermore, investigating its association with long-term outcomes, post-acute sequelae, and recovery patterns may provide insights into its role beyond the acute phase of the disease. Efforts toward standardizing suPAR measurement techniques, establishing cutoff values, and validating its clinical utility as a prognostic marker in larger and diverse patient populations are crucial. This would enhance its reliability and facilitate its integration into routine clinical practice for risk assessment and therapeutic decision-making. Collaborative research efforts involving multidisciplinary teams are pivotal for advancing our understanding of suPAR’s role in COVID-19 coagulopathies. Collaboration between clinicians, researchers, and industry stakeholders is essential for translating research findings into clinical applications and therapeutic interventions.

## Correlation between suPAR and the severity of the above diseases

COVID-19 has been associated with a wide range of complications, including severe respiratory distress, multi-organ failure, and significant alterations in coagulation pathways. Thromboembolic events have been frequently reported in COVID-19 patients, necessitating the need for reliable biomarkers to monitor and manage these complications effectively. suPAR has emerged as a potential biomarker due to its role in inflammation, immune response, and coagulation. The SARS-CoV-2 virus triggers a hyperinflammatory response, leading to cytokine storm and endothelial dysfunction. This results in the activation of coagulation pathways and subsequent thrombotic events. The imbalance between pro-coagulant and anticoagulant factors, along with platelet activation and fibrinolysis suppression, contributes to the coagulopathy observed in COVID-19 patients. suPAR is a circulating form of the uPAR, which is involved in various physiological processes, including fibrinolysis, inflammation, and cell migration. Elevated levels of suPAR have been linked to poor prognosis in infectious diseases and inflammatory conditions. In the context of COVID-19, suPAR levels correlate with disease severity and coagulation dysfunction, making it a valuable biomarker for monitoring disease progression and therapeutic response. Numerous studies have highlighted the correlation between suPAR levels and the severity of COVID-19. Elevated suPAR levels have been associated with worse clinical outcomes, including increased risk of severe respiratory failure, ICU admission, and mortality^[34–36]^.

Higher suPAR levels are linked to greater impairment in respiratory function. For example, studies have shown that patients with elevated suPAR levels are more likely to require mechanical ventilation and have prolonged hospital stays. suPAR levels correlate with other inflammatory markers such as C-reactive protein, IL-6, and ferritin. Elevated suPAR is indicative of a heightened inflammatory state, which is a hallmark of severe COVID-19. suPAR is associated with markers of coagulation dysfunction, such as D-dimer, fibrinogen, and platelet count. High suPAR levels often correspond with elevated D-dimer and fibrinogen levels, as well as thrombocytopenia, reflecting the hypercoagulable state in severe COVID-19. suPAR levels have been shown to correlate with scores measuring organ dysfunction, such as the Sequential Organ Failure Assessment score. Higher suPAR levels are predictive of multiple organ failure and poor prognosis. Several studies have demonstrated that elevated suPAR levels are associated with increased disease severity, ICU admission, and mortality in COVID-19 patients. suPAR can serve as an early indicator of coagulation dysfunction, enabling timely intervention and management of thrombotic complications. Its diagnostic utility extends beyond traditional coagulation markers, providing a more comprehensive assessment of the patient’s coagulation status. Monitoring suPAR levels can guide therapeutic decisions, particularly in anticoagulation therapy. Patients with high suPAR levels may benefit from more aggressive anticoagulation strategies to prevent thrombotic events. Additionally, suPAR could potentially serve as a marker for evaluating the efficacy of anti-inflammatory and immunomodulatory treatments, given its role in the inflammatory cascade. The management of COVID-19-related coagulopathy involves a combination of anti-inflammatory and anticoagulant therapies. Targeting the underlying inflammation with agents such as corticosteroids, tocilizumab, and other immunomodulators can help mitigate the hyperinflammatory state. Concurrently, anticoagulant therapy with heparin or direct oral anticoagulants is crucial in preventing and treating thrombotic events. suPAR levels can assist in stratifying patients and tailoring these therapeutic interventions accordingly^[37,38]^.

## Relevant suPAR-associated pathways or downstream signals can be cited and analyzed for their feasibility as a treatment for DIC caused by COVID-19

COVID-19 has been associated with a wide range of complications, including severe respiratory distress, multi-organ failure, and significant alterations in coagulation pathways. Thromboembolic events have been frequently reported in COVID-19 patients, necessitating the need for reliable biomarkers to monitor and manage these complications effectively. suPAR has emerged as a potential biomarker due to its role in inflammation, immune response, and coagulation. The SARS-CoV-2 virus triggers a hyperinflammatory response, leading to cytokine storm and endothelial dysfunction. This results in the activation of coagulation pathways and subsequent thrombotic events. The imbalance between pro-coagulant and anticoagulant factors, along with platelet activation and fibrinolysis suppression, contributes to the coagulopathy observed in COVID-19 patients. suPAR is a circulating form of the uPAR, which is involved in various physiological processes, including fibrinolysis, inflammation, and cell migration. Elevated levels of suPAR have been linked to poor prognosis in infectious diseases and inflammatory conditions. In the context of COVID-19, suPAR levels correlate with disease severity and coagulation dysfunction, making it a valuable biomarker for monitoring disease progression and therapeutic response[35].

Numerous studies have highlighted the correlation between suPAR levels and the severity of COVID-19. Elevated suPAR levels have been associated with worse clinical outcomes, including increased risk of severe respiratory failure, ICU admission, and mortality. Higher suPAR levels are linked to greater impairment in respiratory function. For example, studies have shown that patients with elevated suPAR levels are more likely to require mechanical ventilation and have prolonged hospital stays. suPAR levels correlate with other inflammatory markers such as C-reactive protein, IL-6, and ferritin. Elevated suPAR is indicative of a heightened inflammatory state, which is a hallmark of severe COVID-19. suPAR is associated with markers of coagulation dysfunction, such as D-dimer, fibrinogen, and platelet count. High suPAR levels often correspond with elevated D-dimer and fibrinogen levels, as well as thrombocytopenia, reflecting the hypercoagulable state in severe COVID-19. suPAR levels have been shown to correlate with scores measuring organ dysfunction, such as the Sequential Organ Failure Assessment score. Higher suPAR levels are predictive of multiple organ failure and poor prognosis. suPAR can activate the NF-κB pathway, leading to the production of pro-inflammatory cytokines such as tumor necrosis factor-alpha, IL-1β, and IL-6. This pathway plays a critical role in the inflammatory response seen in COVID-19. The JAK/STAT signaling pathway, activated by various cytokines, is also influenced by suPAR levels. Elevated suPAR can lead to enhanced activation of this pathway, contributing to the cytokine storm associated with severe COVID-19[39].

suPAR can modulate the activity of the urokinase plasminogen activator, influencing fibrinolysis. High suPAR levels can impair fibrinolysis, contributing to the hypercoagulable state observed in COVID-19. suPAR can interact with integrins on the cell surface, affecting cell adhesion and migration. This interaction is crucial in endothelial function and coagulation. Drugs that inhibit the NF-κB pathway, such as corticosteroids, could help reduce inflammation and cytokine production in COVID-19 patients with high suPAR levels. Agents like baricitinib, which inhibit the JAK/STAT pathway, could potentially reduce the inflammatory response and improve outcomes in patients with elevated suPAR. Low molecular weight heparin is commonly used in COVID-19 to prevent thrombosis. suPAR levels could help identify patients who might benefit from more aggressive anticoagulation strategies. Direct oral anticoagulants such as apixaban or rivaroxaban might be used in patients with high suPAR levels to prevent thromboembolic events^[40–45]^.

## Recommendations

Incorporate suPAR measurements into routine clinical practice, especially for risk stratification and prognostication of COVID-19 patients. Establishing standardized protocols for suPAR assessment could aid in identifying individuals at higher risk of developing severe coagulopathies. Support and conduct further research, including longitudinal studies and clinical trials, to validate the prognostic value of suPAR in diverse patient populations. Investigate its utility in predicting disease progression, severity, and response to therapeutic interventions. Encourage efforts toward standardizing suPAR measurement techniques, assay platforms, and establishing uniform reference ranges. Collaboration among laboratories and researchers is essential to ensure consistent and reliable suPAR measurements across different settings.

Invest in research focused on developing targeted therapeutic interventions that modulate suPAR-associated pathways. Explore the potential of agents or strategies aimed at reducing suPAR levels or mitigating its downstream effects to ameliorate coagulation abnormalities and improve patient outcomes. Develop clinical guidelines and decision-making algorithms integrating suPAR measurements as part of risk assessment and treatment stratification in COVID-19. These guidelines can aid health care providers in making informed decisions regarding patient management and therapeutic strategies.

Implement educational programs and resources to familiarize health care professionals with suPAR’s role in COVID-19 coagulopathies. Training initiatives can help clinicians understand the significance of suPAR measurements and their implications in patient care. Foster collaboration among international research groups, health care institutions, and regulatory bodies to share data, findings, and best practices related to suPAR in COVID-19. Collaborative efforts facilitate a more comprehensive understanding and utilization of suPAR as a biomarker. Ensure adherence to ethical guidelines and obtain informed consent from patients when utilizing suPAR measurements in clinical settings or research studies. Respecting patient autonomy and confidentiality is crucial when incorporating biomarker assessments. Encourage the integration of suPAR measurements into ongoing and future clinical trials and treatment protocols for COVID-19. Assess its utility in predicting therapeutic responses and guiding tailored treatment strategies. Continuously monitor emerging evidence and review updated guidelines regarding suPAR’s role in COVID-19 coagulopathies. Flexibility in adapting clinical practices based on new findings is essential to optimize patient care.

## Conclusion

suPAR stands as a promising biomarker with significant implications in the context of COVID-19-associated coagulopathies. Its role in predicting disease severity, progression, and adverse outcomes in COVID-19 patients has garnered considerable attention, offering insights into potential prognostic value and therapeutic implications. The elucidation of suPAR’s role as a predictive biomarker presents opportunities for improved risk stratification, personalized therapeutic interventions, and enhanced clinical decision-making in managing COVID-19 patients. Integration of suPAR measurements into clinical practice, standardized assay techniques, and collaborative research efforts are imperative for further validating its clinical utility and fostering its translation into routine patient care.

In essence, suPAR holds immense potential as a prognostic marker and a target for therapeutic interventions in COVID-19-associated coagulopathies. The integration of suPAR assessments into clinical guidelines, combined with ongoing research endeavors, offers prospects for advancing our understanding and improving patient outcomes in the evolving landscape of COVID-19 management. Continued exploration of suPAR’s role may pave the way for tailored strategies, ultimately enhancing the care and prognosis of individuals affected by COVID-19 coagulation disorders.

## Data Availability

Not applicable as this a review.

## Research registration unique identifying number (UIN)

Not applicable as this a review.
